# The Use of Facebook Advertising to Recruit Healthy Elderly People for a Clinical Trial: Baseline Metrics

**DOI:** 10.2196/resprot.7918

**Published:** 2018-01-24

**Authors:** Julie M Cowie, Mark E Gurney

**Affiliations:** ^1^ Tetra Discovery Partners, Inc Grand Rapids, MI United States; ^2^ Grand Valley State University Grand Rapids, MI United States

**Keywords:** clinical trial recruitment, medical research, older people, social media, Facebook, research subject recruitment, advertising, elderly

## Abstract

**Background:**

This report provides data on the use of social media advertising as a clinical trial recruitment strategy targeting healthy volunteers aged 60 years and older. The social media advertising campaign focused on enrollment for a Phase 1 clinical trial. Traditional means of recruiting—billboards, newspaper advertising, word of mouth, personal referrals, and direct mail—were not producing enough qualified participants.

**Objective:**

To demonstrate the effectiveness of using targeted advertising on the social networking site Facebook to recruit people aged 60 years and older for volunteer clinical trial participation.

**Methods:**

The trial sponsor used a proactive approach to recruit participants using advertising on social media. The sponsor placed and monitored an Institutional Review Board-approved advertising campaign on Facebook to recruit potential candidates for a Phase 1 clinical trial. The clinical trial required a 10-day residential (overnight) stay at a clinic in Michigan, with one follow-up visit. The sponsor of the clinical trial placed the advertising, which directed interested respondents to a trial-specific landing page controlled by the Contract Research Organization (CRO). The CRO provided all follow-up consenting, prescreening, screening, and enrollment procedures. The campaign was waged over an 8-week period to supplement recruiting by the CRO.

**Results:**

A total of 621 people responded to a Facebook advertising campaign by completing an online form or telephoning the CRO, and the clinical trial was fully enrolled at 45 subjects following an 8-week Facebook advertising campaign.

**Conclusions:**

An 8-week Facebook advertising campaign contributed to 868 inquiries made regarding a Phase 1 clinical trial seeking to enroll healthy elderly subjects. Over the initial 11 weeks of recruitment, 178 inquiries were received using traditional methods of outreach. Respondents to the Facebook advertising campaign described in this report engaged with the sponsored advertising at a higher rate than is typical for social media-based clinical trial recruitment strategies. The older adults’ engagement rate of 4.92% was more than twice as high as click-through rates of younger adults engaged with social media advertising in other clinical trial recruitment studies. Advertising placed on the social media platform Facebook is effective with the healthy volunteer population aged 60 years and older. This approach can quickly and cost-effectively reach qualified candidates for clinical trial recruitment as a supplement to traditional means of recruiting.

**Trial Registration:**

ClinicalTrials.gov: NCT02840279; https://clinicaltrials.gov/ct2/show/NCT02840279 (Archived by WebCite at http://www.webcitation.org/6wamIWXAt)

## Introduction

### Background

Sponsored advertising on social media as a clinical trial recruitment strategy is relatively new. Informed by known barriers to successful enrollment [1,2], Contract Research Organizations (CROs) and sponsors of clinical trials are now using Internet-based outreach to augment traditional ways of recruiting elderly subjects, such as doctor referrals, print advertising, and television advertising [3-5]. In the last few years, there has been increasing research measuring the effectiveness of social media outreach [6], including the use of Facebook [7,8]. Although an increasing number of published articles provide metrics of successful Facebook advertising campaigns [9-11], few discuss Facebook-based recruitment of older adults for participation in clinical trials [12,13].

This report provides an example of clinical trial recruitment of healthy elderly people using Facebook advertising. Older adults are using Facebook in increasing numbers; in 2016, of all online adults, 62% of those aged 65 years and older used Facebook [14]. In 2017, 67% of adults aged 65 years and older said they went online, with 45% of seniors under the age of 75 using social networking sites, along with 20% of those aged 75 and older [15]. There are ample and recent calls to implement social media-based recruitment strategies as an effective and cost-saving approach to clinical trial recruitment [16-19]. The Michael J. Fox Foundation’s Facebook-based recruitment of older Ashkenazi Jews provides an anecdotal success story [20].

This study provides data on an outreach method targeted to healthy elderly adults (age 60 years and over) for enrollment in a Phase 1 multiple ascending dose clinical trial assessing safety, tolerability, and preliminary cognitive benefit of a compound being developed for the treatment of Alzheimer’s disease (NCT02840279). This report presents examples of paid (sponsored) Institutional Review Board (IRB)-approved advertising on Facebook, and the response to the recruitment effort compared to traditional methods. This study demonstrates the cost effectiveness of a targeted advertising campaign over a short duration for a sponsor with no established social media presence prior to the advertising launch.

### Context

The Facebook advertising campaign was launched due to low enrollment by the CRO. The CRO had begun clinical trial recruitment in early June 2016 using the following methods: (1) personal referral; (2) direct mailer (quantity of 6000) sent to surrounding postal ZIP codes, age 60 and older; (3) billboards placed near the clinical site; (4) bus advertising in the city where the clinic is located; (5) newspaper ads in three regional and free “shopper” newspapers; and (6) outreach events.

This outreach, conducted over a period of 11 weeks, resulted in 6 enrolled subjects. The enrollment goal was 45. Due to the low enrollment, three additional strategies for recruitment were implemented: (1) the study fee for the participants was raised from US $2500 to US $4000, (2) outreach from the sponsor increased to include personal contact with leaders of area churches and senior groups, and (3) the sponsor launched a Facebook advertising campaign to direct interested people to the CRO through completion of an online form or by telephone inquiry.

The social media campaign was an intense, immediate, and directed effort to enroll in the trial. The comparative effectiveness of the different social media recruitment strategies against traditional media was made weekly, with adjustment as needed. The Facebook advertising campaign was run by the sponsor, rather than the CRO, and the CRO controlled all contact with respondents. The objective of the Facebook campaign was to enhance awareness and create a trial-unique pathway that allowed potential volunteers to discover and learn more about the clinical trial, and ultimately contact the CRO for further information.

## Methods

### Clinical Trial Design

The clinical trial was a Phase 1 study of a memory drug at a single clinical site. Details of the clinical trial design have been registered at ClinicalTrials.gov (NCT02840279). The study protocol required a 10-day/night stay (residential) in a clinic in Michigan, with one follow-up visit. Participants stayed in dormitory-style rooms, with no visitors permitted. Candidates for the study were required to be nonsmokers, free from any central nervous system medications, with age-normal lab values, well managed diabetes (if diabetic), no history of cancer, healthy blood pressure, and were asked to complete a cognition battery. Recruitment was planned to reflect the demographics of the region surrounding the clinical site, which for Kalamazoo County, Michigan are 51% female and 81.7% white [21].

### Approach

#### Social Media Campaign #1

The initial social media outreach used the same words and images as the traditional campaign. The advertising placed on Facebook used artwork and text that had been approved by the IRB and used for outreach in the prior three months of the recruitment period. The CRO had implemented a recruitment campaign consisting of: billboards, a direct mailing of 6000 postcards sent to local area residents aged 60 years and older, regional newspaper ads, announcement of the trial on the CRO’s website and Facebook page, advertising on buses, recruitment events (including talks at senior centers), and flyers and posters.

**Figure 1:**
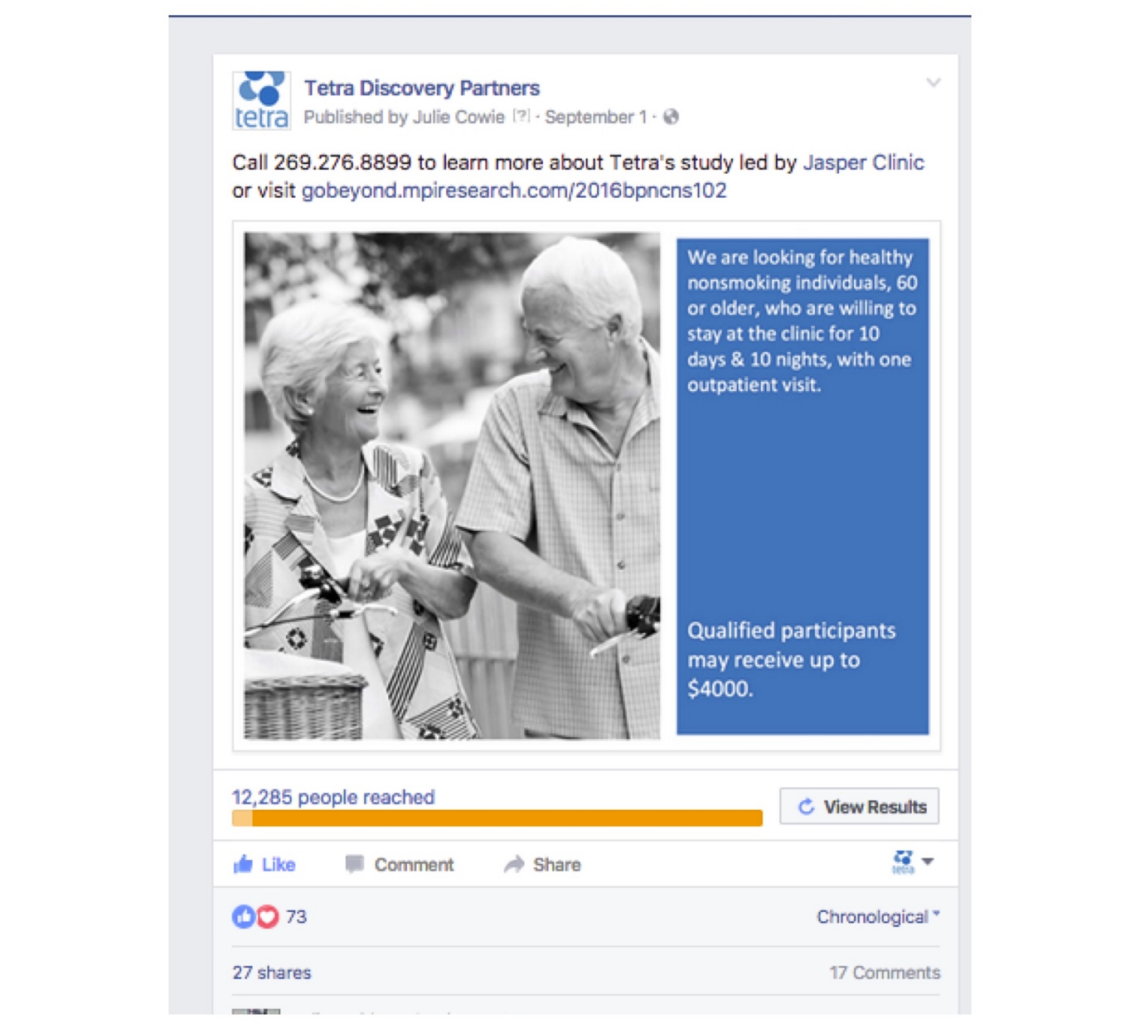
Initial boosted Facebook post using black-and-white image.

The sponsor had no social media presence prior to the start of the trial. Day one of this Facebook advertising campaign consisted of establishing a company page for the sponsor. On the second day of the campaign, Facebook posts were boosted or paid to reach a wider audience. At this early stage of the social media campaign the approach reinforced the advertising already distributed throughout the region. The initial post, a black and white image with text, is shown in Figure 1.

Targeting [22], a tool available to Facebook advertisers, was used to direct this post to Facebook users with the following interests: (1) Alzheimer’s disease research, (2) medical research, and (3) the Alzheimer’s Association. Ads were targeted to individuals aged 60 years and up, with a focus on geographic communities within a 60-minute drive of the clinical site.

The campaign was actively managed, with staff from the clinical trial sponsor monitoring the social media engagement throughout the day and evening. The initial spend was US $150/day for the first four days. Following this initial period, advertising placements and expenditures varied relative to the CRO’s capacity to follow up on inquiries in a timely manner.

Posted comments were acknowledged, usually with a reply to contact the CRO for additional information. Advertising was updated if posted questions or comments suggested that the advertising text was not clear. The CRO provided Health Insurance Portability and Accountability (HIPAA)-compliant feedback to the sponsor about the reasons for failed prescreening. This feedback informed the next iteration of advertising recruitment.

#### Social Media Campaign #2

The sponsor has a track record as an innovative startup, using lean methods [23] to rapidly adjust and experiment as a way of solving problems. In this case, the innovative approach taken was to launch advertising on Facebook and focus on the potential clinical trial participant as a *customer*. The customer segments principle [24] and the use of keywords within Facebook advertising guided the remainder of the advertising campaign.

Two distinct customer segments or Facebook audiences were targeted in the second iteration of the social media campaign. One advertising strategy focused on older adults who would be content with the low level of stimulation a 10-day/night stay would offer and who would be healthy enough to qualify for the study; this was the “typical” campaign. A second advertising strategy was oriented to people who would be altruistically motivated to enroll. This campaign targeted older adults interested in helping to advance scientific progress regarding the treatment of Alzheimer’s disease and memory loss; this was the “altruistic” campaign. Both segments reflect known motivations for participants in clinical trials [25]. The advertising strategy hypothesized that these two customer segments had the distinct attributes shown in Textbox 1.

Facebook advertising provides the ability to target by age, geography, income level, and keywords. These qualifiers were used to narrow the outreach to either the typical or the altruistic customer segments. Keywords can also be used for exclusion. The use of keywords for exclusion narrows the targeted audience even further. The Facebook algorithm seeks to match only one of the keywords, not all. Keywords employed in the advertising campaign are outlined in Table 1.

**Figure 2:**
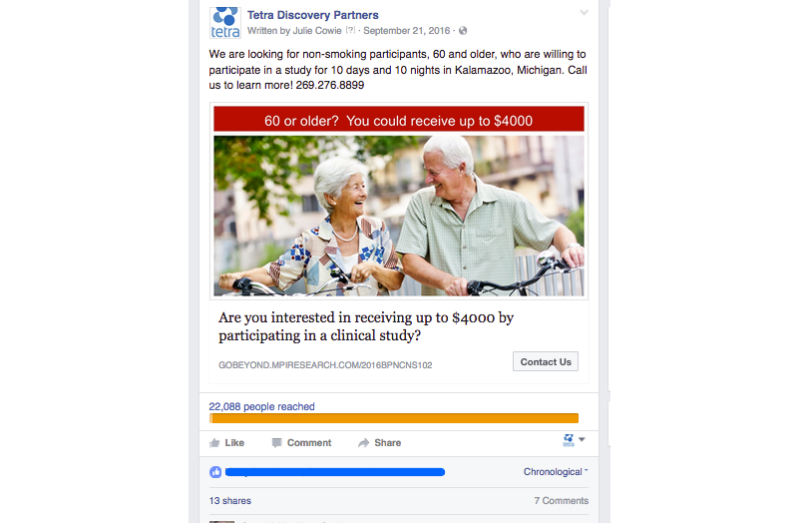
Example of advertising placed for the typical campaign.

**Figure 3:**
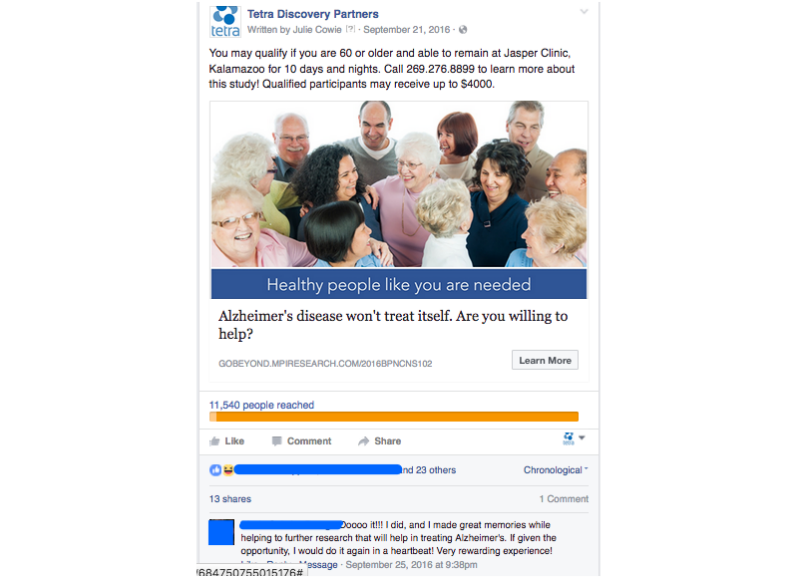
Example of advertising placed for the altruistic campaign.

As the social media campaign evolved, the sponsor submitted for IRB-approval of additional text and photographs more suited to social media. The campaign was configured so that the sponsor paid only for link clicks. All links in the Facebook ad connected to the CRO’s trial-specific landing page. The landing page offered a form to be completed by potential participants expressing interest in the trial. An advertisement representative of the typical campaign is shown in Figure 2. An advertisement representative of the altruistic campaign is shown in Figure 3.

**Description of customer segments.**

Characteristics of the “typical” recruit:

- Sedentary but healthy
- Willing to forego exercise/outdoors for 10 days
- Enjoys television or reading
- Available for a 10-day/night stay with short notice
- Not involved in providing daily care for another

Characteristics of the “altruistic” recruit:

- Civic-minded
- Oriented to philanthropy or religious stewardship
- Motivated to give of oneself and one’s time for a greater good
- Engaged in Alzheimer’s disease awareness or touched by Alzheimer’s disease
- Interested in scientific advancement, medical research, and/or clinical trials

**Table 1:** Keywords used for targeted advertising on Facebook.

Parameter	Typical campaign	Altruistic campaign
Geography	Communities within 90 minutes’ drive of clinical site At or just below median income level for the county (2015 census) Engagements from initial ad were reviewed for geography, with advertising concentrated in communities showing higher rates of engagement	University regions within two hours’ drive Affluent communities within two hours’ drive
Income Level	$100,000 or less	$100,000 or above
Keywords	Clinical trial, Reading, WebMD, Widow, Frugality, Fixed income, Single person, Retirement, Social security, or solitaire	Neuroscience, Clinical trial, Alzheimer's disease research, Philanthropy, Mind games, Costco, Altruism, Medical research, Lumosity, or Lifelong learning
Exclusions	National Cancer Survivors Day, Diabetes mellitus type 2 awareness, Hypertension Awareness, Allergy, Prehypertension, Cancer signs and symptoms, Diabetic diet	National Cancer Survivors Day, Diabetes mellitus type 2 awareness, Hypertension Awareness, Allergy, Prehypertension, Cancer signs and symptoms, Diabetic diet

## Results

The Facebook advertising campaign was conducted over a period of approximately 8 weeks. The campaign concluded when the trial was fully enrolled with 45 subjects.

### Social Media Campaign #1 Results

The initial post, a black and white image with text, received a “1” relevance score on a scale of 1 to 10, with 1 being low. This finding indicated that the ad was not well designed for the target audience [26]. The full set of metrics for the initial ad from social media campaign #1 is shown in Table 2.

In this post, the result rate was 1.9%, representing the ratio of engagements to impressions. Engagements are clicks, likes, shares, or comments. Impressions refers to an ad appearing in a newsfeed. The reach (11,052) represents the number of unique people who viewed the content. Of the 126 unique clicks, approximately 30 online contact forms were completed on the CRO landing page in the first five days. The initial advertising run, which included a three-day holiday weekend, resulted in 27 shares, 73 reactions, and 17 comments. This run also showed cost effectiveness, with a cost per engagement of US $1.23, compared to an industry average for medical campaigns of US $1.32 per click [27]. The clinical site reported results from the media outreach on a weekly basis during the recruitment period. Table 3 shows the results at the end of this initial week of paid Facebook posts.

Despite the high contact rate, most subjects failed the stringent inclusion and exclusion criteria for the study. However, this first iteration of the social media campaign successfully enrolled eight subjects and two alternates in the next dose cohort.

### Social Media Campaign #2 Results

The typical and altruistic campaigns shown in Figure 2 and Figure 3 produced the results shown in Table 4.

The ads from social media campaign #2 used color images, wording specifically chosen for social media, and keyword targeting. This campaign produced click-through rates of 2.79% for the typical campaign and 2.01% for the altruistic campaign.

### Total Campaign Results

Table 5 summarizes the metrics from the entire Facebook advertising campaign.

The overall campaign click-through rate of 3.37% shown in Table 5 exceeded the typical click-through rate range for clinical trial recruitment, which is 0.5-1.2% [28]. The CRO reported the following demographics for participants in the healthy elderly clinical trial, shown in Table 6.

With 29 women and 16 men participating, the study enrolled 64% women. The study overenrolled white subjects. White participants represent 81% of the southwest Michigan population in which the clinical site was located, whereas whites comprise 76.9% of the US population [29].

Table 7 summarizes the total number of inquiries made of the CRO about the trial by outreach form, as reported by the CRO. Of 857 inquiries into the clinical trial, Facebook outreach produced 72.5% (621/857) of them.

**Table 2:** Facebook metrics for initial boosted post shown in Figure 1.

Metric	Total number
Impressions	27,496
Reach	11,052
Link clicks	524
Shares	27
Comments	17
Click-through rate, %	1.9
Cost per click, US$	$1.23
Frequency	2.49
Unique clicks	126

**Table 3:** Summary of responses by medium in the first week of active Facebook advertising. CRO: Contract Research Organization.

Promotional medium	Responses, end of week #1
CRO website	1
Referral	2
Word of mouth	2
Facebook	134
Newspaper ads (3 papers, 3 cities)	6
Billboard	4

**Table 4:** Facebook advertising results for typical and altruistic campaigns.

Metric	Typical campaign	Altruistic campaign
Impressions	44,659	31,080
Reach	22,288	11,488
Link clicks	1246	627
Shares	14	12
Comments	10	4
Click-through rate, %	2.79	2.01
Cost per click, US$	$0.91	$1.27
Frequency	2.0	2.71
Unique clicks	1084	534

**Table 5:** Facebook metrics for advertising campaign targeted to healthy elderly people.

Metric	Total number
Impressions	454,284
Reach	142,228
Link clicks	15,322
Shares	140
Comments	87
Click-through rate, %	3.37
Cost per click, US$	$0.45
Frequency	3.19
Unique clicks	7004

**Table 6:** Demographics of enrolled subjects.

Parameter	Total number
Enrolled subjects	45
Age range	60-78
Number of women	29
Number of men	16
White ethnicity, %	90

**Table 7:** Total inquiries sorted by method of outreach. CRO: Contract Research Organization.

Clinical trial inquiries	Number
CRO website and intranet	81
Facebook	621
Word of mouth/referral/event	64
Print/newspaper ads	61
Poster/flyer/direct mail/billboard	30

## Discussion

The purpose of this report is to demonstrate the effectiveness of using sponsored advertising on Facebook to recruit people aged 60 years and older for clinical trial participation. Initial metrics showed that even with a low relevance score, the initial black and white ad used for social media campaign #1 (and shown in Figure 1) was twice as effective as average health care online advertising. The average click-through rate for health care marketing online is 0.83% [30] and this result rate was 1.9%. Moreover, the Facebook advertising tool proposed a potential reach of 7000, and the actual reach of 11,052 exceeded this estimate by over 50%. Analysis of social media campaign #2 by gender shows that women slightly favored the altruistic campaign, and men favored the typical campaign. Results are shown in Table 8.

When considering the whole campaign, the engagement rate of men was slightly higher than the engagement rate of women, meaning that men were more likely to click on the advertisement than women. The advertising appeared to more women than men, with Facebook reporting that 71% of the impressions were to women. The results in Table 9 show this distinction of click activity by gender.

Facebook advertising can be a cost-effective method to recruit people aged 60 years and older into Phase 1 clinical trials. Respondents to the Facebook advertising campaign described in this report engaged with the sponsored advertising at a higher rate than younger adults engaged with social media advertising in other clinical trial recruitment studies [31].

In Table 10, metrics for this study are contrasted to two others: one involving young adults up to age 25 for a smoking cessation intervention [32]; and one aimed at young women, aged 16-25, regarding sexual health [21]. In contrast to people of younger ages, sponsored advertising for this campaign geared to healthy people aged 60 years and above prompted a notably high proportion of unique clicks to campaign reach. This finding affirms what other researchers have shown: people aged 55-64 are twice as likely to engage with sponsored Facebook advertising than younger adults [31].

The amount of commenting and sharing also exceeded typical standards. This advertising campaign received positive comments (posted on more than one ad) from a person who had completed the study. The effects of this are immeasurable and certainly rare [28]. The ads were monitored several times per day throughout the campaign, with most comments receiving some kind of timely acknowledgment or reply. Negative comments were unusual but did occur.

Minority enrollment in the study was not proportional to the US population and lagged behind the demographics of the population surrounding the clinical site. Reasons for low enrollment are not known but may relate to the demographics of Facebook users regionally, the images used for the Facebook ad campaign (which predominantly depicted white ethnicity subjects), and the gap in recruitment rates of minorities when recruiting older people in general [4].

**Table 8:** Comparison of responses by gender, and typical and altruistic campaigns.

Response		Women	Men
**Typical campaign**			
	Reach, n (%)	15,344 (69.48)	6740 (30.52)
	Clicks, n (%)	827 (70.74)	342 (29.26)
	Click-through rate, %	5.38	5.07
**Altruistic campaign**			
	Reach, n (%)	10,116 (88.92)	1260 (11.08)
	Clicks, n (%)	587 (94.22)	36 (5.78)
	Click-through rate, %	5.80	2.86

**Table 9:** Engagement and cost by gender for the Facebook advertising campaign.

Total campaign	Women	Men
Impressions, n (%)	322,185 (71.52)	128,266 (28.48)
Reach, n (%)	98,721 (70.04)	42,227 (29.96)
Cost, total US$ (%)	5200 (76.81)	1570 (23.19)
Cost per click, US$	$0.0526	$0.0371
Engagement rate, %	3.06	3.29

**Table 10:** Comparison of engagement rates for advertising targeted to elderly and younger adults.

Age groups	Campaign reach	Unique clicks	Clicks per reach (%)	Cost per click (US$)	Overall cost (US$)	Number of subjects needed
Healthy Age 60+	142,228	7004	4.92	$0.45	$6828	45
Smokers age 18-25	961,131	5895	0.61	$0.34	$2024	230
Females age 16-25	469,678	7940	1.69	$0.67	$5400 (estimated)	200

**Table 11:** Inquiries made of a clinical trial, with and without Facebook advertising.

Recruitment period	Number of weeks^a^	Facebook advertising campaign	Inquiries from all advertising^b^	Enrolled subjects
June 13 - August 28	11	No	178	6
August 29 - October 25	8	Yes	691	39

^a^Approximated.

^b^Results provided by Contract Research Organization.

Other gaps in data stem from the relationship between the CRO and the sponsor. The Facebook advertising campaign was initiated by the sponsor, without extensive coordination with the CRO. The recruitment process was parallel but distinct, and specific recruitment data tracked by the CRO was not shared with the sponsor. In this study, the following are not known: how many people completed the online form compared to telephoning their interest, how many of the 621 responses attributed to the Facebook campaign were contacted for prescreening, the subjects’ precise reasons for enrolling in the study, and how much money was spent by the CRO on more traditional forms of recruitment. Data that showed how the enrolled subjects learned of the clinical trial opportunity was not provided to the sponsor, so a cost-per-compliant participant from this Facebook campaign cannot be ascertained.

In this discussion, a sponsor with no prior presence on Facebook completed recruitment for a single-site, Phase 1 clinical trial following a Facebook advertising campaign. The Facebook advertising was used in addition to other forms of outreach and demonstrated effectiveness in recruiting qualified candidates, as shown in Table 11.

This study showed that interest in (and response to) a clinical trial focused on healthy elderly participants can be increased through a targeted Facebook advertising campaign.

### Conclusion

Results from this Facebook advertising campaign show that a sponsor who placed advertising on Facebook targeted to healthy people aged 60 years and older prompted enough interest in the clinical trial to successfully recruit a full cohort in a period of less than two months, thereby closing the gap created by clinical trial recruitment outreach using traditional methods alone.

## Abbreviations

CRO: Contract Research Organization
IRB: Institutional Review Board
